# Isolation, Whole-Genome Sequencing, and Annotation of Two Antibiotic-Producing and -Resistant Bacteria, Enterobacter roggenkampii RIT 834 and Acinetobacter pittii RIT 835, from Disposable Masks Collected from the Environment

**DOI:** 10.1128/mra.00757-22

**Published:** 2022-09-12

**Authors:** Angeline R. Mozrall, Renata Rezende Miranda, Grish Kumar, Crista B. Wadsworth, André O. Hudson

**Affiliations:** a Pittsford Sutherland High School, Pittsford, New York, USA; b The Gosnell School of Life Sciences, Rochester Institute of Technology, Rochester, New York, USA; University of Arizona

## Abstract

We report the whole-genome sequence and annotation of two antibiotic-resistant bacteria, Enterobacter roggenkampii RIT 834 and Acinetobacter pittii RIT 835, isolated from disposed masks. We found that these strains are resistant to five of seven commonly used antibiotics and that they produce bactericidal compounds against Escherichia coli.

## ANNOUNCEMENT

**FIG 1:**
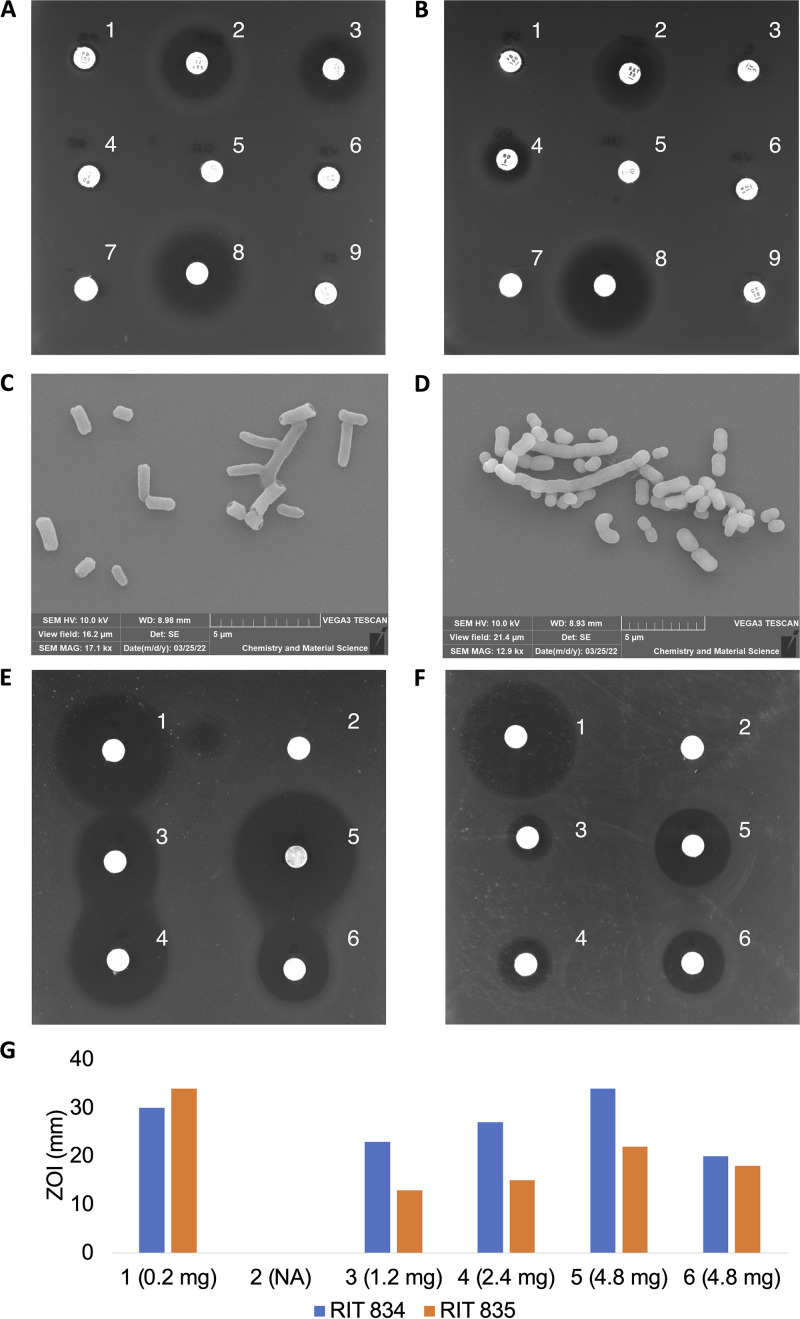
Disk-diffusion susceptibility tests of *E. roggenkampii* RIT 834 (A) and A. pittii RIT 835 (B), each treated with polymyxin B, 300 IU (1); sulfamethoxazole/trimethoprim, 25 μg (2); chloramphenicol, 30 μg (3); rifampicin, 5 μg (4); clindamycin, 2 μg (5); vancomycin, 30 μg (6); methanol, 20 mL (7); tetracycline, 200 μg (8); and colistin sulfate, 10 μg (9). (C and D) Scanning electron micrographs (SEM) showing Enterobacter *roggenkampii* RIT 834 cells (C), 1.0 to 2.0 m in diameter (magnification, ×17,100), and Acinetobacter pittii RIT 835 cells (D), 1.0 to 2.0 m in diameter (magnification, ×12,900). Disk-diffusion inhibitory assays of *E. roggenkampii* RIT 834 (E) and A. pittii RIT 835 (F) spent R2A medium extracts tested against Escherichia coli ATCC 25922: tetracycline, 200 μg (1); methanol, 20 mL (2); 1,200 μg (3), 2,400 μg (4), and 4,800 μg (5) spent R2A medium extract crude; and 4,800 μg (6) blank R2A medium (no bacteria) extract crude. (G) Bar graph illustrating the results shown in panels E and F. Comparison between the zone of inhibition (ZOI) values and the increasing amounts of crude extracts of *E. roggenkampii* RIT 834 and A. pittii RIT 835. NA, not applicable.

The COVID-19 pandemic has led to a global increase in the use of disposable masks and other personal protection equipment (1). This practice poses risks not only to the environment by increasing pollution from discarded masks, but also to human health, since masks are potential reservoirs for resistant bacteria that may be pathogenic (2). To evaluate the risk of bacterial resistance spread through disposable masks, we isolated, sequenced, and annotated the genomes of Enterobacter roggenkampii RIT 834 and Acinetobacter pittii RIT 835 from surgical masks collected from the environment near the Rochester Institute of Technology (RIT) on 3 February 2022. Enterobacter species are generally considered human pathogens (3), and *E. roggenkampii* in particular has been shown to harbor antibiotic resistance genes (4). Small squares of four different masks were incubated in tryptic soy broth (TSB) and Reasoner’s 2A (R2A) broth at 30°C under aerobic conditions, followed by dilution and plating. Four distinct colonies were chosen from each mask for an antibiotic susceptibility screen against seven commonly used antibiotics. Both *E. roggenkampii* RIT 834 and A. pittii RIT 835 were isolated from the same mask and selected for further studies due to their high antibiotic resistance profiles (Fig. 1A and B). They form white colonies on R2A agar and upon electron microscopy examination (5), show both individual and clumps of rod-shaped cells, about 1 to 2 μm in diameter (Fig. 1C and D). Ethyl acetate spent medium extracts of both bacteria were tested for antimicrobial activity using a disk diffusion inhibitory assay (6) against Escherichia coli ATCC 25922 (Fig. 1E and F). *E. roggenkampii* RIT 834 showed higher bactericidal activity than A. pittii RIT 835 (Fig. 1G).

Genomic DNA (gDNA) was isolated from a 5-mL single colony cultured in R2A medium using the GenElute bacterial genomic DNA isolation kit (Sigma-Aldrich, USA) according to the manufacturer’s protocol. For sequencing, the gDNA was quantified using a NanoDrop spectrophotometer. The library preparation for Illumina sequencing was performed using the Nextera XT library preparation kit (Illumina Inc., USA) following the manufacturer’s instructions. The fragment size distribution was checked using a high-sensitivity DNA kit on an Agilent 2100 Bioanalyzer. The libraries were quantified using a Qubit 3.0 fluorometer and diluted to 16 pM. Sequencing was performed on an Illumina MiSeq (v3 chemistry, 2 × 300 cycles) instrument at the Genomics Lab at RIT.

The demultiplexed FASTQ files were processed for quality control using the program fastp (7). We performed *de novo* assembly of the filtered data using Unicycler v0.5.0 (8), which uses SPAdes v3.15.4 (9) to assemble the short reads. The results of the genome assembly are presented in Table 1. The quality of the genome assemblies was assessed using QUAST (10), and all metrics were computed for contigs that exceeded 500 bp. The assignment of genomes to genera and species was performed using a fast bacterial genome identification platform (fIDBAC; http://fbac.dmicrobe.cn/). We used the combination of genome-wide average nucleotide identity (gANI) and alignment fraction (AF) with a cutoff of 96.5% (gANI) and 0.6 (AF) for species assignment. The genomic sequencing reads were annotated using the Prokaryotic Genome Annotation Pipeline (PGAP), which is a component of the Read Assembly and Annotation Pipeline Tool (RAPT; https://www.ncbi.nlm.nih.gov/rapt). We present the results of the annotation in Table 1.

**TABLE 1:** Sequencing, assembly, and annotation results for the Enterobacter roggenkampii RIT 834 and Acinetobacter pittii RIT 835

Characteristic	Data for strain:	
	Enterobacter roggenkampii RIT 834	Acinetobacter pittii RIT 835
GenBank accession no.	JANFCY000000000	JANFCX000000000
SRA accession no.	SRR19504269	SRR19504268
Assembly size (bp)	4,866,590	4,026,825
No. of reads	3,988,812	5,939,348
No. of contigs	195	139
Coverage (×)	239	402
*N*_50_ (bp)	104,028	93,821
Assembly GC content (%)	55.81	38.85
No. of coding genes	4,664	3,853
No. of tRNAs	74	67
No. of rRNAs	1	2
%gANI^*a*^	98.65	97.17
AF^*b*^	0.86	0.85

a gANI, genome-wide average nucleotide identity.

b AF, alignment fraction, a measure of the proportion of genes shared with reference assemblies in the NCBI database.

### Data availability.

The whole-genome assemblies and Sequence Read Archive (SRA) accession numbers for the bacterial genomes are presented in Table 1 and available for download through GenBank and the SRA, respectively.
